# Increasing Threat of Brucellosis to Low-Risk Persons in Urban Settings, China

**DOI:** 10.3201/eid2001.130324

**Published:** 2014-01

**Authors:** Shouyi Chen, Hao Zhang, Xiaoning Liu, Wenjing Wang, Shuiping Hou, Tingting Li, Shuoxian Zhao, Zhicong Yang, Chengyao Li

**Affiliations:** Center for Disease Control and Prevention, Guangzhou, China (S. Chen, H. Zhang, X. Liu, S. Hou, Z. Yang);; Southern Medical University, Guangzhou, China (W. Wang, T. Li, S. Zhao, C. Li)

**Keywords:** brucellosis, bacteria, human infection, public health, urban setting, risk, China

## Abstract

Cases of brucellosis were diagnosed in 3-month-old twins and their mother. An epidemiologic survey suggested that raw sheep or goat meat might be the source of *Brucella melitensis* infection. This finding implies that the increasing threat of brucellosis might affect low-risk persons in urban settings in China.

Brucellosis, a zoonotic disease, causes severe pain and impairment in humans. In 2012, the Chinese Center for Disease Control and Prevention (China CDC) reported 39,515 new cases of human brucellosis, and this number is increasing by 10% each year. Generally, brucellosis is associated with persons who are occupationally in contact with *Brucella* spp.–infected animals or products (*1*,*2*). However, in this report, we present a cluster of cases of brucellosis in a family living in Guangzhou, China. These data illustrate a trend of human brucellosis threatening theoretically low-risk persons in an urban setting and suggest a need for eradicating or controlling *Brucella* spp.–infected animals and products in China.

## Case Reports

Congenital brucellosis was diagnosed in patients 1 and 2, who were 3-month-old twins (Technical Appendix Table). They were prematurely delivered by cesarean section on July 6, 2012, at the Provincial Maternity and Child Care Center (Guangzhou, China). The boy (patient 1, Apgar score 9–10/1–10 min) had a birthweight of 2.3 kg, and the girl (patient 2, Apgar score 9–10/1–10 min) had a birthweight of 1.8 kg. They received standard care for preterm neonates at the hospital. They were discharged once their weight reached 2.5 kg; this happened for patient 1 at 3 weeks of age and for patient 2 at 4 weeks of age (July 29 and August 3, 2012, respectively).

On October 2, 2012, the boy was examined at the hospital for irregular fever up to 39°C. On October 9, he was readmitted to the hospital with a fever of 38°C and weight of 5.0 kg. Chest radiograph showed signs of increased bronchovascular shadows. Mezlocillin sodium and sulbactam sodium (4:1) and ribavirin were administered, but the patient did not improve. On the same day, the girl had a cough and low-level fever (37–37.5°C) but was not hospitalized. On October 16, *B. melitensis* was isolated from a blood culture from patient 1, in whom brucellosis with alveobronchiolitis, abnormal hepatic function, and moderate anemia were initially diagnosed when he was 3 months and 10 days of age.

On October 17, the twins were transferred to an infectious disease hospital, where they had extensive physical and laboratory examinations (Table 1). During 57 days of hospitalization, the boy received general and specific therapies for brucellosis. Brucellosis and glucose-6-phosphate dehydrogenase deficiency were diagnosed in the girl, and she received appropriate treatment. At the time of discharge (December 12), the twins were well and without fever. They left the hospital for home care, which was supervised by a local general practitioner who provided rifampin and sulfamethoxazole for up to 6 weeks.

**Table 1:** Clinical and laboratory data on twin patients on admission to the infectious disease hospital

Variables*	Patient 1, twin boy	Patient 2, twin girl	Reference range (children)†
Temperature, °C	38	37.0	36–37
Pulse, beats/min	128	128b	120
Respiratory rate, breaths/min	34	36	30–35
Blood pressure, mmHg	76/42	76/45	80/48
Weight, kg	5.7	5.6	
Erythrocyte count, cells/L	4.99 × 10^12^	4.69 × 10^12^	3.5–5.5 × 10^12^
Hemoglobin, g/L	85	89	120–160
Hematocrit, %	26.3	27.5	40–50
Erythrocyte sedimentation rate, mm/h	5	No record	<10
Leukocyte count, cells/L	7 × 10^9^	8.51 × 10^9^	4.0–10.0 × 10^9^
Differential count, %			
Neutrophils	18.8	10.7	50–75
Eosnophils	3	1.1	0.5–5
Basophiles	0.2	0.3	0–1.0
Lymphocytes	74.3	83.9	20–40
Monocytes	3.7	4	3.0–10.0
Platelet count, per L	462 × 10^9^	456 × 10^9^	100–300 × 10^9^
Sodium, mmol/L	103	135.4	135–145
Potassium, mmol/L	33	No record	3.4–4.8
Glucose 6-dehydrogenase, U/L	4,254	1,363	≥2,500
Lactate dehydrogenase, U/L	356	322	100–380
Alanine aminotransferase, U/L	31	48	5–40
Aspartate aminotransferase, U/L	54	64	5–40
Alkaline phosphatase, U/L	508	642	30–390
Total bilirubin, µmol/L	8.49	5.17	5.10–22.2
Total protein, g/L	51	49	60–68
Albumin, g/L	37	40	35–55
Globulin, g/L	14	9	20–35
C-reactive protein, mg/L	0.2	0.21	0.03–5
Creatine kinase, U/L	49	203	24–194
Creatine kinase-MB, U/L	32	25	0–25
*Brucella* antibody titer, SAT	400	800	<100
Blood culture	*B. melitensis*	*B. melitensis*	
Hepatitis B surface antigen	Negative	Negative	
Antibody to hepatitis B surface antigen, IU/L	6.77	Negative	
Hepatitis B e antigen	Negative	Negative	
EBV IgA	Negative	Negative	
Cytomegalovirus IgM	Negative	Negative	
Herpes simplex virus 1, 2 IgM	Negative	Negative	
Influenza virus A + B antigens	Negative	Negative	
*Mycoplasma* IgM	Negative	Negative	
*Chlamydia pneumoniae* IgM	Negative	Negative	
*Toxoplasma* IgM	Negative	Negative	

*Major items are presented from clinical testing. EBV, Epstein-Barr virus. †The ranges used at this hospital are not all for children, and may not be appropriate for the twin patients. The values may be affected by the laboratory methods in different hospitals.

Patient 3 (the mother of patients 1 and 2), a 31-year-old woman who was admitted to a hospital on July 4, 2012 for threatened premature labor at 34 weeks and 2 days’ gestation. Chorioamnionitis phase I was diagnosed that day. On July 6, the patient gave birth to twins through a uterine lower segment cesarean section due to early rupture of the amniotic membrane. Postnatally, the mother was in generally good clinical condition without specific complaint and was discharged for home care on July 11. When *Brucella* infection was diagnosed in her son (patient 1), she was hospitalized for suspected *Brucella* infection (Technical Appendix Table). Brucellosis was diagnosed, and she was treated as an outpatient with a 4-week course of rifampin and doxycycline and 1-week course of streptomycin. Her symptoms of brucellosis rapidly improved.

Serologic and bacteriologic tests were conducted for diagnosis of *Brucella* infection. On October 17, 2012, blood samples were taken from all 6 members of the patients’ family. By standard tube agglutination test, the twins and their mother tested positive for *Brucella* antibodies with titers of 400 (twins) and 800 (mother), whereas results for the twins’ father and grandparents were negative. *Brucella* antibodies from the twins’ blood samples were detected 3 times with titers of 400, 200, and 200 on November 10, 18, and 29, respectively. Plasma from the twins’ cord blood tested positive by a rose bengal plate test, but results were indeterminate or negative by standard tube agglutination test (titer <50). Samples from patients 1–3 were collected on October 17, and on October 25, after 8 days of blood cultures on commercial agar plates 3 *Brucella* strains were isolated. A *Brucella* sp. was repeatedly isolated in blood samples collected on November 10 but not in samples collected on November 18 and 29, after the patients were treated with rifampin. The mother’s breast milk was collected before and after she was treated for brucellosis, and *Brucella* sp. was not isolated from these samples.

**Figure 1:**
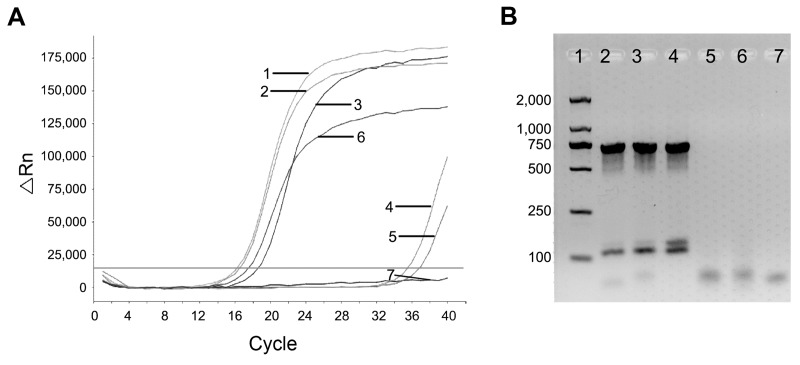
Detection and identification of *Brucella* DNA. A) Detection of *Brucella* DNA by quantitative PCR. Numbers indicate amplification curves with cycle threshold (C_t_) values representative of samples. Curve 1, sample from patient 1 with 16 C_t_ value; curve 2, sample from patient 2 with 16 C_t_; curve 3, sample from patient 3 with 17 C_t_; curve 4, stem cells of cord blood from patient 1 with 34 C_t_; curve 5, stem cells of cord blood from patient 2 with 34.5 C_t_; curve 6, positive control with 18 C_t_, curve 7, negative control with no C_t_. B) Amplification of *Brucella* DNA by AMOS-PCR. Numbers indicate the amplified DNA bands representative of samples. Lane 1, DNA molecular weight marker, values along the left side are base pairs; lane 2, sample from patient 1; lane 3, sample from patient 2; lane 4, sample from patient 3; lane 5, stem cells from cord blood of patient 1; lane 6, stem cells from cord blood of patient 2; lane 7, negative control.

*Brucella* DNA was tested by quantitative PCR of blood cultures from 3 patients, from patients’ cord blood, and from a positive control (Figure 1, panel A). Additionally, the specific DNA bands for *B. melitensis* were identified from each patient’s blood culture by an abbreviated *B.* *abortus*, *melitensis*, *ovis*, and *suis* (AMOS) PCR (*3*) but were not observed from the twins’ cord blood, possibly due to low levels of *Brucella* DNA (Figure 1, panel B).

**Figure 2:**
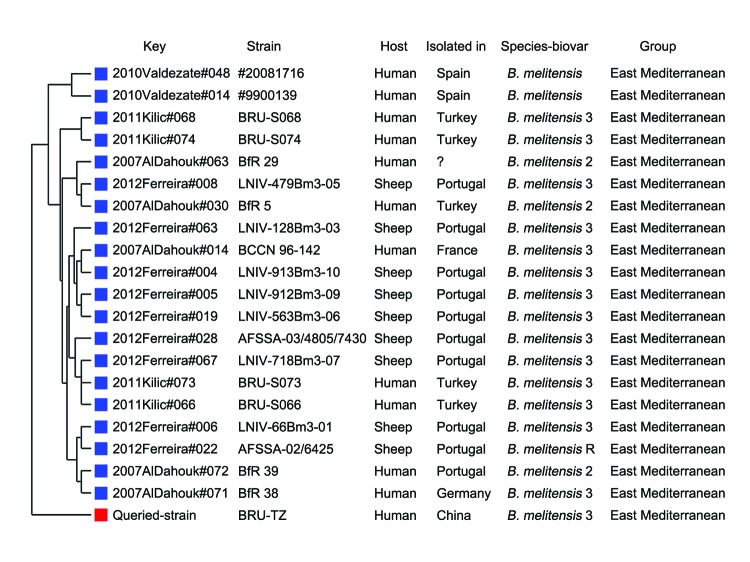
Genetic relationship between the strain isolated in this study (BRU-TZ) and other *Brucella melitensis* strains. The variable number of tandem repeats were obtained for phylogenetic analysis at multiple-locus variable-number tandem repeat analysis bank version-4 (http://mlva.u-psud.fr) (*5**,**6*). The phylogenetic tree was plotted on the differences in variable number of tandem repeats at 16 loci obtained by multiple-locus variable-number tandem repeat analysis.

Bacterial isolates were characterized as *B. melitensis* biotype 3 (Table 2) (*4*). By multilocus variable-number tandem repeat analysis of 16 samples (*5*), these *Brucella* isolates from the twins and their mother were genetically identical. They were all genotyped as 16 loci, with variable number of tandem repeats of 1 5 3 13 2 3 3 2 6 22 9 6 9 11 4 5, which was phylogenetically closer to #20081716 and #9900139 strains prevalent in Spain (Figure 2) but differed from strains prevalent in Kyrgyzstan (*6*).

**Table 2:** Bacteriological and biochemical features of *Brucella* strains

Strain	TZ (twin boy)	TS (twin girl)	ML (mother)
CO_2_ requirement	–	–	–
H_2_S production	–	–	–
Dye inhibition*			
Thionin	+	+	+
Basic fuchsin	+	+	+
Mono-specific anti-serum agglutination†			
A	+	+	+
M	+	+	+
R	–	–	–
Lysis test by *Brucella* spp. phage‡			
Tb10^4^	–	–	–
Tb	–	–	–
Wb	±	±	±
BK_2_	+	+	+
Identification			
Species	*B. melitensis*	*B. melitensis*	*B. melitensis*
Biovar	3	3	3

*A final concentration of 20 μg/mL dyes was used in the testing (*4*). †The bacterial isolate was tested for agglutination by mono-specific anti-serum samples to *Brucella* antigens A, M, and R (rough), respectively. ‡Bacterial isolate was tested for lysis by specific *Brucella* phages of Tb, Wb, and BK2.

## Conclusions

Patients with brucellosis usually have occupations that involve interaction with animals or clinical or laboratory veterinary work. There are reports of human brucellosis related to blood transfusion (*7*), bone marrow transplantation (*8*), transplacental transmission (*9*), breast feeding (*10*), or sexual activity (*11*). In this study, a cluster of brucellosis was identified in 3 patients from a 6-member family. However, the mother and other family members denied having risk factors associated with brucellosis. During the mother’s pregnancy, she had fever and aching bones, while the grandmother occasionally prepared steamed stuffed buns containing raw sheep or goat meat, which reportedly were bought at the supermarket or local butcher’s shop. Raw meat might therefore constitute the source of the *Brucella* infection.

In recent years human brucellosis cases have spread quickly from rural to urban areas and increased sharply in persons in China who do not fit into standard risk categories. Guangzhou, a major city in southern China, is located far away from the *Brucella-*endemic areas of northern China but has recorded increasing numbers of human brucellosis: >60 cases in the past 5 years (China CDC, unpub. data). Live animals and raw meat products are frequently transported across the whole country, and cases of brucellosis have been recorded in all regions of the country (*12*). About 85% of brucellosis cases have been attributed to *B. melitensis* from infected sheep or goats (*12*,*13*), which put ordinarily low-risk persons at much higher risk when they consumed or handled infected animal meat and milk (*14*). The increasing numbers of cases of brucellosis indicates that the strategy of vaccination and quarantine for infected animals has failed in China. One possible reason is the limited efficacy of the current vaccines (*2*,*15*), but a primary reason is that the policies for eradication and control of *Brucella-*infected animals and their products have not been adequately implemented.

Technical Appendix Time points of clinical examination for the 3 patients with brucellosis reviewed in this article.
