# Supplementary material for: Increasing Threat of Brucellosis to Low-Risk Persons in Urban Settings, China
Source: Emerg Infect Dis. 2014 Jan;20(1):126–30. doi: 10.3201/eid2001.130324 (PMC3884711; doi:10.3201/eid2001.130324)
Supplement: Technical Appendix — Time points of clinical examination for the 3 patients with brucellosis reviewed in this article. [file 13-0324-Techapp-s1.pdf]

Article DOI: http://dx.doi.org/10.3201/eid2001.130324

Increasing Threat of Brucellosis to Low-Risk Persons in Urban Settings, China

Technical Appendix

Technical Appendix Table. Time points of clinical examination for 3 patients with brucellosis*

Date (in 2012)	Twin boy (patient 1)	Twin girl (patient 2)	Mother (patient 3)
July 4			Admission to maternity and child care center with premature labor at 34 wk and 2 d of gestation
July 6	Delivered by cesarean section; birth weight is 2.3 kg	Delivered by cesarean section; birth weight is 1.8 kg	
July 11			Discharged
July 29	Discharged for home care weighing 2.5 kg		
August 3		Discharged for home care weighing 2.5 kg	
October 2	Outpatient at hospital for irregular fever up to 39°C		
October 9	Re-admission to hospital for fever up to 38°C	Outpatient at hospital for cough and low fever 37°C–37.5°C	
October 16	*Brucella* sp. isolated		
October 17	Transferred to infectious disease hospital.<br>SAT antibody titer 400	Admission to infectious disease hospital.<br>SAT antibody titer 400	Outpatient at infectious disease hospital.<br>SAT antibody titer 1:800
October 25	*Brucella* sp.isolated	*Brucella* sp. isolated	*Brucella* isolated
November 10	*Brucella* sp. isolated; SAT antibody titer 400	*Brucella* sp. isolated; SAT antibody titer 400	*Brucella* isolated
November 18	*Brucella* sp. not isolated; SAT antibody titer 200	*Brucella* sp. not isolated; SAT antibody titer 200	*Brucella* not isolated
November 29	*Brucella* sp. not isolated; SAT antibody titer 200	*Brucella* sp. not isolated; SAT antibody titer 200	*Brucella* not isolated
December 14	Discharged for home care supervised by local GP	Discharged for home care supervised by local GP	Discharged for home care supervised by local GP

*SAT, standard tube agglutination test; GP, general practitioner.
